# Mitochondria as a Source and a Target for Uremic Toxins

**DOI:** 10.3390/ijms20123094

**Published:** 2019-06-25

**Authors:** Vasily A. Popkov, Denis N. Silachev, Arthur O. Zalevsky, Dmitry B. Zorov, Egor Y. Plotnikov

**Affiliations:** 1 A.N. Belozersky Institute of Physico-Chemical Biology, Moscow State University, Moscow 119992, Russia; popkov.vas@gmail.com (V.A.P.); silachevdn@belozersky.msu.ru (D.N.S.); 2 V.I. Kulakov National Medical Research Center of Obstetrics, Gynecology and Perinatology, Moscow 117997, Russia; 3 Faculty of Bioengineering and Bioinformatics, Lomonosov Moscow State University, Moscow 119992, Russia; aozalevsky@fbb.msu.ru; 4 Sechenov First Moscow State Medical University, Institute of Molecular Medicine, Moscow 119991, Russia; 5 Shemyakin-Ovchinnikov Institute of Bioorganic Chemistry of the Russian Academy of Sciences, Moscow 117997, Russia

**Keywords:** uremia, oxidative stress, mitochondria, kidney injury, toxins

## Abstract

Elucidation of molecular and cellular mechanisms of the uremic syndrome is a very challenging task. More than 130 substances are now considered to be “uremic toxins” and represent a very diverse group of molecules. The toxicity of these molecules affects many cellular processes, and expectably, some of them are able to disrupt mitochondrial functioning. However, mitochondria can be the source of uremic toxins as well, as the mitochondrion can be the site of complete synthesis of the toxin, whereas in some scenarios only some enzymes of the pathway of toxin synthesis are localized here. In this review, we discuss the role of mitochondria as both the target and source of pathological processes and toxic compounds during uremia. Our analysis revealed about 30 toxins closely related to mitochondria. Moreover, since mitochondria are key regulators of cellular redox homeostasis, their functioning might directly affect the production of uremic toxins, especially those that are products of oxidation or peroxidation of cellular components, such as aldehydes, advanced glycation end-products, advanced lipoxidation end-products, and reactive carbonyl species. Additionally, as a number of metabolic products can be degraded in the mitochondria, mitochondrial dysfunction would therefore be expected to cause accumulation of such toxins in the organism. Alternatively, many uremic toxins (both made with the participation of mitochondria, and originated from other sources including exogenous) are damaging to mitochondrial components, especially respiratory complexes. As a result, a positive feedback loop emerges, leading to the amplification of the accumulation of uremic solutes. Therefore, uremia leads to the appearance of mitochondria-damaging compounds, and consecutive mitochondrial damage causes a further rise of uremic toxins, whose synthesis is associated with mitochondria. All this makes mitochondrion an important player in the pathogenesis of uremia and draws attention to the possibility of reducing the pathological consequences of uremia by protecting mitochondria and reducing their role in the production of uremic toxins.

## 1. Introduction

Chronic kidney disease (CKD) is a potentially life-threatening complication of kidney pathologies that affects more than 10% of the human population (more than 850 million people) worldwide. CKD is characterized by loss of kidney function beyond three months, which includes impaired excretion of various waste products of metabolism and endogenous and exogenous toxins. Obviously, such a disorder leads to accumulation of a myriad of compounds in the circulation, and many of them are toxic or at the very least disrupt normal organism functioning in different ways. The cumulative effect of such disturbances in kidney excretion and subsequent toxicity is called “uremic syndrome” and can be classified with up to 75 individual clinical symptoms, which can affect every organ [1,2]. The principal clinical manifestations of uremia were summarized by Almera and Argiles [2] and include disorders of the memory and cognitive functions, asthenia, headache, confusion, anorexia, gastroparesis, hematologic anemia, hemostasis disorders, hypertension, atherosclerosis, coronary artery disease, skin itching, skin dryness, calciphylaxis, growth impairment, impotence, infertility, sterility, osteomalacia, b2-microglobulin amyloidosis, increased sensitivity to infections, metabolic acidosis, hyperphosphatemia, hyperkaliemia, and many others. The serious consequences of uremia include cardiovascular diseases as they are the leading causes of death among CKD patients [3].

While uremia has clearly recognized clinical symptoms, elucidation of the molecular and cellular mechanisms of disease progression is a very challenging task. This is because more than one hundred molecules are now considered to be “uremic toxins” [4], thus making it extremely difficult to analyze their diverse effects on different tissues and cell types and the possibility of interplay of such effects. To this end, the European Uremic Toxins (EUTox) workgroup is an international consortium of academic and medical researchers, which have dedicated their work to studying uremic toxins and everything related to uremia. The outcomes have been impressive as they have described at least 136 molecules that are considered to be uremic toxins. On closer observation, it is emerging that uremic toxins seem to be a very diverse group of molecules that can be described using different classifications, none of which are ideal. The conventional classification includes three distinct groups of uremic toxins based on their physico–chemical properties: the small water-soluble compounds, the protein-bound solutes, and the middle (0.5–60 kD) size molecules [4]. However, the chemistry of uremic toxins is very different, and it includes peptides, ions, products of lipid and carbohydrate oxidation and products of nucleotides modification, etc.

CKD, uremia, and the pleiotropic effects of uremic toxins are so vast and diverse in affecting most of the organs and signaling pathways that even up to the present day there is no widely accepted “synthetic understanding” of the patho-physiology of CKD. This is due to the comprehensive mechanisms of this pathology being rather “mosaic” and in a fragmented state. Some researchers compared this field with the poem titled “The blind men and the elephant” by John Godfrey Saxe, where blind people try to describe an elephant in possession of limited information [5]. In addressing this issue, Himmelfarb and colleagues proposed the idea that oxidative stress could offer a framework in which these pathologies could be described and analyzed systemically [5]. They (as well as other researchers) developed this approach further [6,7]. However, we believe that the emphasis should not be made on oxidative stress *per se*, but rather on mitochondrial functions, which are tightly associated with oxidative stress. Thus, in this review, we discuss the novel role of the mitochondrion in the context of both the target and source of pathological pathways and compounds prevalent in uremia, which may present a novel paradigm and offer new avenues of investigation.

## 2. Mitochondria in Acute and Chronic Kidney Pathologies

**Figure 1:**
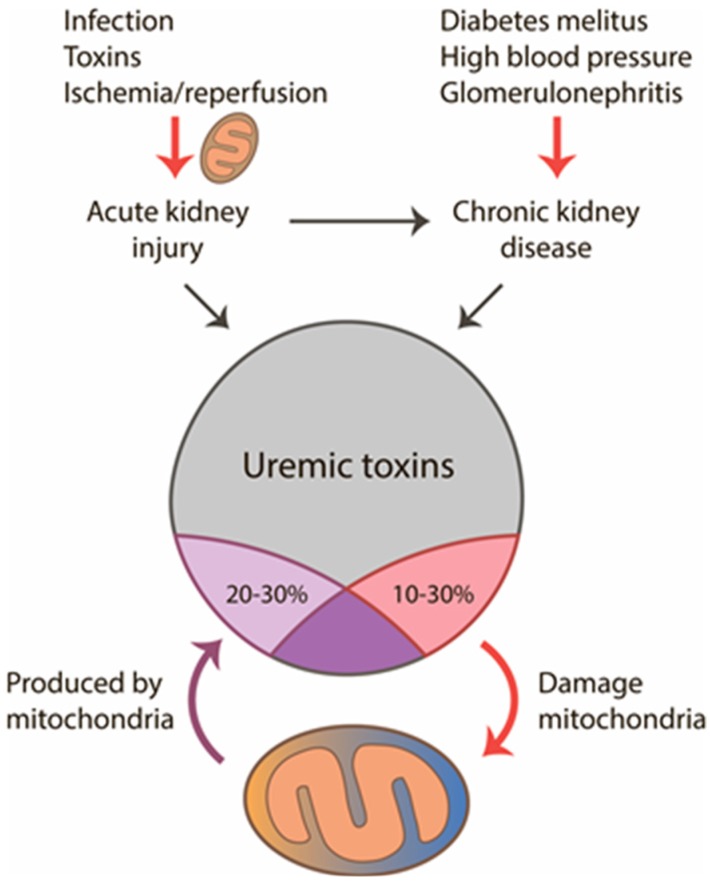
Interaction of mitochondria with uremic toxins in acute and chronic renal pathologies. Mitochondria are involved in the development of acute kidney injury (AKI), and later when uremia develops, mitochondria can act as a source and target for uremic toxins. From all uremic toxins, up to 30% could be produced by mitochondria and about 20% have damaging effects on the organelles.

It is well established and described that mitochondria play a key role in the development of acute kidney injury (AKI) arising from different pathogenesis, such as ischemic, infectious, or toxic (Figure 1). This role is not uni-directional as mitochondria are one of the main targets of the initial damaging stimulus, such as ischemia or toxic drugs, and one of the main sources of secondary damage as well. Here, when mitochondrial structure and functions are disturbed, they become the main source of pathological reactive oxygen and nitrogen species (ROS and RNS, respectively) [8], thus amplifying oxidative stress [9] and mediating damaging effects through inflammation, cell death, etc. In most cases, the emergence of CKD is dependent on mitochondria and oxidative stress-related mechanisms as well [10,11,12]. CKD is associated with some initial pathologic event, which provokes impairment of renal functions. Many such CKD-provoking pathologies are related to mitochondria dysfunction and oxidative stress [13], as in AKI [14,15], diabetes [16], hypertension [17], glomerulonephritis and infections [18], skeletal muscle loss, and decreased exercise endurance [19]. In main AKI-provoking pathologies such as ischemic kidney injury or sepsis, the key initial events include mitochondria membrane damage, transmembrane potential drop, opening of mitochondrial permeability transition pore, and release of various pro-apoptotic factors from mitochondria, ultimately leading to renal cell death. Reasonably, mitochondria targeted approaches, such as use of mitochondria-targeted antioxidants, afford protection from AKI and ultimately decrease probability of CKD development [18]. In its turn, CKD can ignite oxidative stress and mitochondrial damage [20,21,22]. The indirect effect of mitochondria on the morbidity in CKD is also expressed in the fact that cardiovascular diseases as the main cause of death during CKD and uremia are also tightly associated with mitochondrial damage and oxidative stress [23,24]. Pathologies of the central nervous system, such as neuropathy, neurotoxicity, blood–brain barrier injury, and neuroinflammation, are also associated with oxidative stress [25,26,27] stemming from mitochondrial dysfunction. Thus, oxidative stress and impaired mitochondrial functioning play a major role in all stages of disease development during AKI, CKD, and uremia to their comorbidities. However, the relationship between oxidative stress, mitochondria, and uremic toxins is yet to be extensively defined.

## 3. Mitochondria as a Source of Damaging and Toxic Molecules

As more than 130 uremic toxins have been described [28], it is therefore not surprising that some of these can affect normal mitochondrial function. However, mitochondria can be the source of uremic toxin production too, thus linking function and production of uremic toxins closely with mitochondrial metabolism, probably via key biochemical pathways. Lastly, some of them are oxidation products of some cellular compounds that are linked to mitochondrial ROS during disease development. Thus, using biochemical pathway maps, we have identified some mitochondrial metabolic pathways strongly associated with uremic toxin synthesis (Table 1). The analysis revealed that among all uremic toxins, more than 20% are either completely synthesized in mitochondria under normal conditions, or at least are closely associated with mitochondrial metabolic pathways (Figure 1). All mitochondria-related uremic toxins can be divided into 2 large groups. The first group includes molecules that are related to normal mitochondrial metabolism, such as those synthesized in mitochondria or are closely associated with mitochondrial metabolic pathways. The second group includes excessive products derived from the pathological oxidation of cellular components by mitochondrial ROS, which are not produced in large quantities under normal conditions.

### 3.1. Enzymatically Produced Compounds

One of the most well-known uremic toxins is creatinine (or closely related creatine), which is a product of the creatine kinase/phosphocreatine system. Creatinine and creatine metabolism are tightly associated with mitochondria (comprehensively reviewed in [29]) and provide reversible conversion of creatine into phosphocreatine within mitochondria and the cytosol. This cycle serves as a buffer for ATP levels, which is especially important in tissues with high-energy demands and is one of the main components of energy homeostasis. Mitochondrial creatine kinase is the key enzyme for this process and directly determines the levels of creatine and creatinine in uremic serum.

Eight organic acids, which can represent another group of uremic toxins, the metabolism of which are dependent on mitochondria, include argininic acid, hippuric acid, indole-3-acetic acid, orotic acid, α-keto-δ-guanidinovaleric acid, γ-guanidinobutyric acid, uric acid, and kynurenic acid. Orotic acid is a component of pyrimidine metabolism and can be synthesized by dihydroorotate dehydrogenase, which is an authentic mitochondrial enzyme [30]. Argininic acid, α-keto-δ-guanidinovaleric acid, and γ-guanidinobutyric acid are members of arginine metabolism (as well as other uremic toxins) and thus are highly dependent on fully-functioning mitochondria and are key players in arginine metabolism. Enzymes such as amidinotransferase, d-amino-acid oxidase, and *N*-acetyl-l-glutamate synthase, which are described as being involved in the synthesis or metabolism of these acids, are also localized in mitochondria [31,32,33]. Hippuric acid is a product of phenols and phenylalanine metabolism and can be produced by mitochondrial glycine N-benzoyltransferase [34]. Indole-3-acetic acid is involved in tryptophan metabolism and can be produced by mitochondrial aldehyde dehydrogenase [35]. Lastly, kynurenic acid is an intermediate of the kynurenine system, which metabolizes L-tryptophan to NAD^+^. Additionally, NAD^+^ is the key coenzyme of basic redox reactions essential for mitochondrial functioning, thus the kynurenine-3-monooxygenase, located in the outer mitochondrial membrane, makes mitochondria an important source of kynurenic acid and component of general metabolism [36].

Uric acid, xanthine, and hypoxanthine, which are linked to uremic toxins, are closely connected with mitochondrial metabolism and are moreover associated with cardiac injuries and numerous oxidative stress-dependent pathologies [37,38]. They are also involved in purine metabolism within mitochondria, as xanthine oxidase catalyzes the conversion of hypoxanthine to xanthine and then conversion of xanthine to uric acid [37].

Another closely related metabolite included in the list of uremic toxins is urea. In all mammals, it is the main end-product of protein metabolism and ammonia detoxification and its synthesis is associated with the mitochondria through the so-called urea cycle. Although two enzymes of this cycle are located in mitochondria (carbamoyl phosphate synthetase I and ornithine transcarbamoylase), urea itself is produced in the cytosol by arginase I. The main site of urea synthesis is located in the liver, but there is evidence of significant urea synthesis by mitochondrial arginase II in the renal tubular cells [39].

Uremic toxins also include several nucleotide derivatives, and at least a few of them can be potentially modified inside the mitochondria. Here, N_2_,N_2_-dimethylguanosine is modified by N_2_,N_2_-dimethylguanosine-specific tRNA methyltransferase in the cytoplasm and mitochondria [40], pseudouridine is modified by pseudouridine synthase in mitochondria as well as in the cytoplasm [41], and 1-methylguanosine is modified by tRNA-(m(1)G37)-methyltransferase in the cytoplasm and mitochondria [42]. Moreover, 8-hydroxy-2′-deoxyguanosine is the product of DNA oxidation, primarily in the mitochondria [39].

Lastly, some enzymatically modified amino acids belong to the group of uremic toxins. Here, *S*-adenosylhomocysteine is catalyzed by *S*-adenosylhomocysteine hydrolase [43], and asymmetric dimethylarginine (ADMA) is generated in mitochondria where proteins with dimethylated arginine residues are degraded [34]. Symmetric dimethylarginine (SDMA) can be further metabolized in mitochondria [44]. However, the majority of amino acids during uremia are modified non-enzymatically, as in the case of sugars, lipids, and nucleotides, which are described in the next section.

Among the toxins, the synthesis of which are related to the mitochondria (Table 1), phenylacetylglutamine is worth mentioning, due to it being a toxin with blood concentrations in excess of more than 100 times the normal during CKD, the levels of which are used as a mortality index [32].

### 3.2. Oxidation Products: Advanced Glycation and Lipoxidation End Products (AGE and ALE) and Aldehydes

As described above, many molecules that are considered as uremic toxins can be synthesized by mitochondria via enzymatically catalyzed reactions. However, even larger numbers of compounds can be generated non-enzymatically (or consequently by the enzymatic detoxication of such molecules), most of which originate from the unintended oxidation of cellular components by ROS. Since mitochondria are believed to be the main source of ROS in the cell; the production of such uremic toxins can be closely linked to mitochondrial metabolism.

In the context of uremia, a few types of oxidatively modified molecules are of most importance, namely, “reactive carbonyl compounds” (RCO), advanced glycation end-products (AGE), and aldehydes. These compounds are formed through a variety of mechanisms and pathways and include numerous precursors. They can have both endogenous and exogenous origins and are very hard to strictly classify due to interconversion reactions. In simplified terms, interaction with ROS and RCO leads to the formation of AGE proteins (with covalently modified sugar degradation products) and advanced lipoxidation end-products (ALE), which are covalent adducts of RCO and produced during lipid peroxidation [46]. Notably, RCO production and “carbonyl stress” are highly dependent on ROS production, “oxidative stress”, and thus on mitochondrial function [47,48].

AGE, ALE, and RCO can modify proteins non-enzymatically and impair their normal functions, which can obviously have the potential to modulate every single process in the cell. It was shown that levels of such compounds rise during uremia, including but not limited to those that are considered uremia toxins themselves [49,50,51,52]. Among uremic toxins, glyoxal and methylglyoxal, pentosidine, carboxymethyllysine, 2-nonenal, 4-HO-nonenal (4-HNE), malondialdehyde (MDA), 2-nonenal, 4-HO-hexenal, 3-deoxyglucosone, 2-hexenal, hexanal, nonanal, 2-octenal, and heptanal can be included in this highly heterogeneous group. However, due to complex classification criteria and due to a high degree of interconversion potential, many other uremic toxins can also be included in this list, especially several aldehydes described as uremic solutes, which are products of redox reactions similar to those occurring during ALE/AGE formation.

Methylglyoxal and glyoxal are believed to be the two key molecules in this group, which belong to both ALE and AGE. Methylglyoxal can be produced during lipid peroxidation, glycolysis, and threonine catabolism. In the latter case, it is produced by monoamine oxidase from 3-aminoacetone and there is evidence of sub-mitochondrial localization of this enzyme [53]. Using mass spectrometry, both glyoxal and methylglyoxal were seen to localize in mitochondria, which additionally proves that AGE and ALE production is indeed linked to mitochondrial function [54].

One of the best-known ALE is 4-HNE, a product of lipid oxidation, which is considered to be one of the most critical and dangerous compounds generated during oxidative stress, and which disrupts signal transduction and protein activity and triggers cellular apoptosis. Ultimately 4-HNE is metabolized by mitochondria [55].

Additionally, mitochondria are involved in detoxication and metabolism of a number of uremic toxins, amongst which there are many aldehydes, such as nonanal, 2-heptenal, 2-hexenal, 2-octenal, 4-decenal, 4-HO-decenal, 4-HO-octenal, decanal, heptanal, methylglyoxal, hexanal, 4-HO-hexenal, and 2-nonenal. Potentially, all of these can be metabolized by mitochondrial aldehyde reductase [35]. Additionally, putrescine, kinurenine, and dimethylglycine can be metabolized by diamine oxidase [56], kynurenine 3-monooxygenase [57], and dimethylglycine dehydrogenase [58,59], respectively, all of which are mitochondrially located. Moreover, symmetric dimethylarginine has also been reported to be metabolized in mitochondria [44].

## 4. Mitochondria as a Target for Uremic Toxins

In addition to energy production, the mitochondrion serves as an important hub that regulates many processes in the cell. While the key cause of kidney mitochondria damage in the development of AKI and CKD has been discussed above, the uremic consequences of AKI and CKD also have fundamental importance in the functioning of mitochondria. In this case, it is possible to speculate not only about mitochondria damage in the kidney but how uremia may also affect other tissues. Consequently, some toxins that affect mitochondria functions are produced by mitochondria, which can create a dangerous self-amplifying loop of toxin production.

For example, 4-HNE, which was already mentioned as a product of lipid oxidation, can disrupt mitochondrial metabolism in many ways [60]. 4-HNE is seen to impair ATP production and even trigger apoptosis [61], which might be attributed to its ability to uncouple mitochondria [62]. One of the main effects of 4-HNE is the non-specific modification of different molecules. It has the potential to disrupt almost every single process in the cell. Obviously, redox-homeostasis-related molecules like thioredoxin [63], Nrf2, peroxisome-proliferator-activated receptors (PPAR), mitochondrial aldehyde dehydrogenase 2 (ALDH2), nuclear factor-κB (NF-κB), and apoptosis-promoting proteins, including activating protein-1 (AP-1) and mitogen-activated protein kinases (MAPK), are also among its targets [63,64,65,66,67,68]. All these numerous effects lead to a wide spectrum of pathological phenotypes caused by 4-HNE, most of which are mitochondria-centered, such as cancer [69], Alzheimer’s disease [70], Parkinson’s disease [71], heart diseases [72,73], diabetes [74], and lung injuries [75]. Several studies have shown that 4-HNE and MDA level rises correlate with other uremic toxin levels. It is debatable whether 4-HNE and MDA levels increase as a result of uremia or as a result of oxidative stress [76].

The most extensively studied mitochondria-affecting uremic toxins can interact with a hydrophobic moiety of proteins, thus providing the modification of a variety of mitochondrial proteins. Deleterious effects that can be induced in mitochondria by such uremic toxins as indoxyl sulfate, indole acetic acid, p-cresol (pC), p-cresyl sulfate (pCS), hippuric acid, kynurenic acid, and 3-carboxy-4-methyl-5-propyl-2-furanpropanoic acid (CMPF) are well-described.

Indoxyl sulfate (IS) is an albumin-bound uremic toxin that has gained attention because of its inability to be removed by dialysis due to its binding to serum proteins with high affinity [77] and thus its accumulating in the serum of CKD patients [78]. Indoxyl sulfate is an end product of dietary tryptophan metabolism produced in the colon by bacterial tryptophanase with further oxidation and sulfonation in the liver [79]. Normally, indoxyl sulfate is a substrate for organic anion transporters localized in the basolateral membrane of proximal tubular cells and can be excreted into the urine via active tubular transport [80]. Indoxyl sulfate has a wide toxic effect not only on kidney tubular cells but also on cells from other organs, including muscle, [19] heart [81], brain [82], lung [83,84], and vascular endothelium [85,86]. We believe that one of the main targets of indoxyl sulfate toxicity is the mitochondria, which explains the non-specific toxic nature of the damage it induces in various organs. The involvement of mitochondria in the development of indoxyl sulfate intoxication has been shown in a number of studies. Treatment with indoxyl sulfate reduced the expression of PGC-1α, decreased mitochondrial membrane potential, and induced autophagy in C2C12 cells [87], whereas co-incubation with various antioxidants such as ascorbic acid, L-carnitine, or teneligliptin restored these parameters to their original state. Similar results were obtained in a rat subtotal-nephrectomy model, where the damage of skeletal muscle was associated with an increase in plasma concentration of indoxyl sulfate. It has been shown that CKD induced downregulation of genes involved in mitochondrial biogenesis and significantly increased superoxide production in the skeletal muscle [88]. Similarly, many studies demonstrated that the uremic solute indoxyl sulfate enhanced ROS production in several cell types [89,90,91]. Study of uremic cardiomyopathy model using H9c2 cells confirmed the mitochondrial origin of ROS induced by indoxyl sulfate [81]. However, treatment of HUVEC cells with indoxyl sulfate resulted in NADPH oxidase-dependent ROS generation, since the inhibitor of NADPH oxidase, diphenyleneiodonium (DPI), decreased indoxyl sulfate-induced ROS production [92]. These inconsistencies were partly explained by simultaneous production of excessive ROS by both NADPH oxidase and mitochondria in endothelium in parallel with RhoA/ROCK activation organizing a positive reciprocal relationship to induce endothelial dysfunction through impaired endothelium-dependent NO signaling. The inhibitor of NADPH oxidase–apocynin, mitochondria-targeted antioxidant Mito-Tempo, and antioxidant TEMPOL restored the levels of markers of oxidative stress to a norm with Mito-TEMPO being more effective than TEMPOL, indicating a key role of mitochondria-derived ROS upon indoxyl sulfate stimulation [85]. We also need to take into account that DPI, being an inhibitor of NADPH oxidase potently inhibits mitochondrial ROS production [93].

It is well-known that ROS could be the main cause of mitochondrial fission [94,95]. Therefore, evidence linking indoxyl sulfate with mitochondrial fission (reviewed in [96]) is of particular importance. This study revealed modulation of the expression of mitochondrial fission/fusion proteins and reduced mitochondrial mass by activating autophagic machinery after indoxyl sulfate treatment, which impaired aerobic and anaerobic metabolism in vivo and in vitro. Similar effects were demonstrated for another uremic toxin, p-cresol sulfate, where antioxidant treatment protected cells from mitochondrial dysfunction and impaired biogenesis [96]. Mitochondrial ROS production was demonstrated for A549 cells (adenocarcinoma human alveolar basal epithelial cells) treated with p-cresyl sulfate, as measured by the MitoSOXTM probe, while mitochondria-targeted antioxidant Mito-TEMPO diminished ROS levels [84]. Moreover, o−, m−, and p-cresols induced a dose-dependent decline in the State 3 respiration rate in isolated rat liver mitochondria, and affected NAD- and succinate-dependent respiration [97] while having little effect on the P/O ratio. Moreover, o-, m-, and p-cresols inhibited uncoupled mitochondrial respiration and accelerated the swelling of liver mitochondria, suggesting that liver mitochondria may be one of the targets for the hepatotoxic actions of cresols.

One of the main targets for uremic toxins in mitochondria is the electron transport chain. The respiratory chain consists of several multi-protein complexes with active centers having different redox potentials, which can be a target for many chemical agents. In turn, the inhibition of the respiratory chain by various compounds can lead to excessive production of pathological ROS [98,99]. A number of diseases have been identified as being caused by mitochondrial dysfunction, and many pharmaceuticals have been subsequently identified as previously unrecognized mitochondrial toxicants. A much smaller but growing amount of literature indicates that environmental pollutants can also target mitochondria [100].

Exposure of conditionally immortalized human renal proximal tubule epithelial cells to uremic toxins (p-cresol, p-cresyl sulfate, and p-cresyl glucuronide) inhibited mitochondrial succinate dehydrogenase activity by more than 20% as measured by 3-(4,5-dimethylthiazol-2-yl)-2,5-diphenyltetrazolium bromide (MTT) reduction. However, uremic toxins putrescine and oxalate did not significantly decrease MTT reduction indicating no noticeable effects on complex II. Moreover, indole-3-acetic acid decreased the maximal respiration rate by 18% in State 3 (using p-trifluoromethoxyphenylhydrazone (FCCP)) [28]. Later, Ellis and colleagues also showed that indoxyl sulfate decreased mitochondrial function (MTT reduction) in primary proximal tubule cells and HK-2 cells [101].

Hippurate is a protein-binding toxin that is mainly present in plant food and normally exists in the human serum at concentrations below 5 mg/L. In patients with CKD, hippurate serum concentrations dramatically increase and can become as high as 471 mg/L [102]. Hippurate, as a uremic toxin, has been reported to be involved in uremia-associated cardiovascular diseases [103]. Treatment of human aortic endothelial cells with hippurate reduced the expression of endothelial nitric oxide synthase and increased the expression of intercellular cell adhesion molecule-1 and von Willebrand factor. The mechanisms of hippurate-induced endothelial dysfunction in vitro depended on the activation of dynamin-related protein 1 (Drp1)-mediated mitochondrial fission and overproduction of mitochondrial ROS. Hippurate affects some mitochondrial enzymes as well, such as inhibition of ammonia production by P-dependent mitochondrial glutaminase, which is primarily stimulated by metabolic acidosis. Simultaneously, hippurate stimulates P-independent glutaminase localized at the proximal luminal membrane, thus shifting the ammonia production from mitochondria to the proximal tubular lumen. Metabolic acidosis, in turn, stimulates hippurate synthesis in the liver and kidney, increases its plasma concentration, and creates a positive regulatory loop of ammoniagenesis [104].

Another uremic toxin, kynurenic acid, provides a link between CKD and heart pathologies through mitochondrial functioning, since this substance affected the respiratory parameters of heart mitochondria in a dose-dependent manner. In the presence of 125 μM kynurenic acid, a significant decrease of respiratory control and ADP/O ratio of rat heart mitochondria were observed using glutamate/malate as substrates rather than succinate [105]. Kynurenic acid lowers the efficacy of mitochondria ATP synthesis, significantly increasing State IV respiration, and the effects were dose-dependent, especially at higher kynurenic acid concentrations (625 μM to 10 mM), where the measured parameters changed dramatically. Metabolites of kynurenic acid such as anthranilic acid, 3-hydroxykynurenine, and 3-hydroxyanthranilic acid also demonstrated a negative effect on the respiratory parameters of heart mitochondria [106].

Above, we have considered the effect of uremic toxins on mitochondria; however, CKD is associated with not only impaired excretion of endogenous uremic toxins but also with accumulation of some widespread exogenous pollutants and toxins originating from protein catabolism by intestinal bacteria, for example, bisphenol A. Bisphenol A is a ubiquitous environmental toxin structurally related to p-cresol, and although bisphenol does not belong to uremic toxins, it significantly accumulates in the blood of CKD patients. Bisphenol A, in a dose-dependent manner, increased depolarization of mitochondria from human renal proximal tubular epithelial cells (HK-2 cell line) at a concentration of 200 μM. In addition, bisphenol A increased the intracellular release of Ca^2+^ and mitochondrial ROS-production assessed by Fura-2 and MitoSox probes, correspondingly [107]. Chronic exposure of rats to bisphenol A for 2 weeks caused mitochondrial dysfunction in liver cells expressed in decreased activities of mitochondrial electron-transport chain complexes I, II, III, IV, and V with a significant increase in mitochondrial superoxide generation [108].

## 5. Uremia, Oxidative Stress, and Antioxidant Treatment

Since it has been extensively proven that oxidative stress and mitochondria are a major factor in CKD and uremia, it is possible to recommend mitochondria-targeted approaches for treatment, the most obvious being the administration of antioxidants. Indeed, there is some evidence that the pool of antioxidants is diminished during CKD, such as glutathione and glutathione peroxidase levels in plasma, which were seen to be decreased in [109]. The activities of antioxidative enzymes, superoxide dismutase (SOD), and glutathione peroxidase were reported to be decreased in patients undergoing hemodialysis [110], as well as the levels of such antioxidants as retinol [111], vitamin C [112], lycopene, delta-tocopherol, gamma-tocopherol, and hydrogen sulfide, were reduced in hemodialysis patients [111,112,113,114]. Indoxyl sulfate, uremic solute, was also shown to enhance ROS production, increase NAD(P)H oxidase activity, and decrease glutathione levels in endothelial cells [92].

It is important to note that a decrease in antioxidants can be linked not only to oxidative stress but to hemodialysis procedures and a specific diet during CKD as well. Irrespectively, depletion of antioxidants during oxidative-stress dependent pathology represents a potentially dangerous situation. Moreover, iron therapy, which is prescribed to hemodialysis patients, can potentially worsen the pro-oxidative status even further [115]. Therefore, there have been various attempts to use antioxidants to treat CKD using vitamins C [116] and E [117], L-carnitine [118,119], omega-3 fatty acids [120], and Q10 [121], which all had some effect on plasma concentration of oxidative stress markers. N-acetylcysteine decreased uremia-induced atherosclerosis in an animal CKD model [122]. It also had a positive effect on the hematocrit as well as on the levels of different oxidative stress markers [123,124]. In an randomized controlled trial including 134 patients, it was also proven that N-acetylcysteine reduce cardiovascular events in patients with CKD undergoing hemodialysis [125].

A meta-analysis [126] revealed that antioxidant therapy significantly reduced the development of end-stage kidney disease, lowered serum creatinine levels, and improved creatinine clearance, but showed no clear overall effect on cardiovascular mortality, all-cause mortality, cardiovascular disease, coronary heart disease, cerebrovascular disease or peripheral vascular disease. Antioxidant therapy was found to significantly reduce the development of end-stage kidney disease, lowered serum creatinine levels, and improved creatinine clearance. Authors of the study emphasized that beneficial effects of antioxidant were based on a small number of events and required more data to reliably assess the effect of antioxidant therapy. We hypothesize that these antioxidant-based approaches fail to provide strong positive results because of imprecise targeting. If mitochondria are one of the key components in uremia toxin production, we should focus on mitochondria-targeted therapeutic approaches, such as mitochondrial targeting by antioxidants MitoQ [127] and SkQ [128], mitochondrial permeability transition pore inhibitors (Li ions [129], cyclosporine [130]), and mitochondria fission inhibitors (MDVI [131]).

## 6. Conclusions

**Figure 2:**
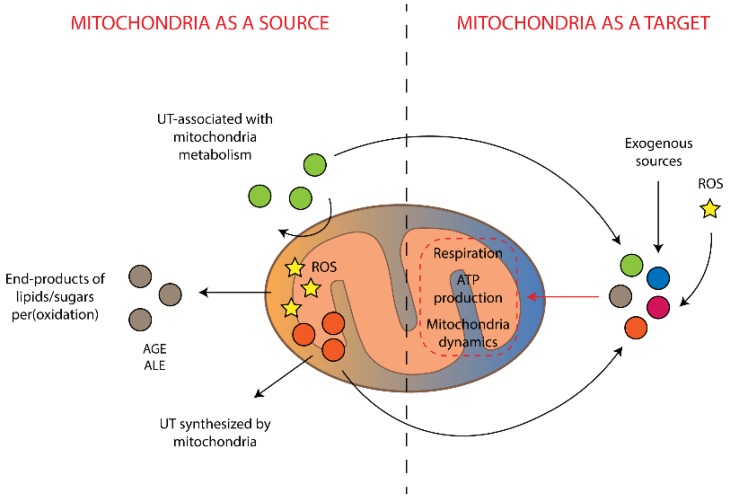
Interrelations between mitochondria-associated production of uremic toxins and damaging effects of the toxins on mitochondria. UT is uremic toxins, AGE is advanced glycation end-products, ALE is advanced lipoxidation end-products, ROS is reactive oxygen species.

To some extent, a number of pathways for uremic toxins synthesis are associated with mitochondria. In one case, the mitochondrion is the site of direct synthesis of the toxin; in another, some enzymes of the metabolic pathways of toxin synthesis are localized in mitochondrion. Since the mitochondrion is a key regulator of cellular redox homeostasis and may be associated with the development of oxidative stress, its functioning directly affects the production of uremic toxins associated with oxidation of cellular components, such as aldehydes, advanced glycation end-products, advanced lipoxidation end-products, and reactive carbonyl species. In addition, a number of metabolic products can be degraded by the mitochondria, and mitochondrial dysfunction is expected to accumulate these products in the organism as uremic toxins. It is important to note that many uremic toxins (produced in mitochondria, appearing from other sources or having exogenous origin) are damaging to mitochondrial proteins, especially the respiratory chain complexes (summarized in Figure 2). As a result, it is possible for a positive feedback loop to arise, leading to the amplification of accumulated uremic solutes. Therefore, CKD can lead to the appearance of mitochondria-damaging compounds and subsequent mitochondrial damage can cause further accumulation of uremic toxins, whose syntheses are associated with mitochondria. This makes the mitochondrion an important player in the pathogenesis of uremia and draws attention to the possibility of reducing the pathological consequences of CKD by protecting mitochondria through reducing their production of uremic toxins.

The main limitation of our analysis is yet undeveloped and fragmentary knowledge of metabolic pathways. The KEGG database is a very useful tool, but it is not complete and does not contain some enzymatic reactions, which are mentioned in PubMed. However, we consider that this limitation is not critical for our analysis.

## Figures and Tables

**Table 1:** Bioinformatically identified uremic toxins, which are metabolically connected to mitochondria. Pathways were identified through the analysis of the KEGG database [45]. Enzymes are highlighted in italic, enzymes that have a mitochondrial localization are highlighted in bold, and arrows indicate direction of reaction.

Toxins	Metabolic Pathway
Creatine	Phosphocreatine ← ***^Creatine kinase, mitochondrial (CKMT1B/CKMT1A/CKMT2)^*** → Creatine
Orotic acid	Dihydroorotate ← ***^Dihydroorotate dehydrogenase (DHODH)^*** → Orotic acid
Hippuric acid	Benzoyl-CoA← ***^Glycine-N-acyltransferase^*** → Hippuric acid
Methylglyoxal	Aminoacetone ← ***^Monoamine oxidase A/B (MAOA/MAOB)^*** → Methylglyoxal
α-Keto-δ-guanidinovaleric acid	Arginine ← ***^D-amino acid oxidase (DAO)^*** → α-keto-δ-Guanidinovaleric acid
Urea	Arginine ← ***^Arginase 1 (ARG1)^*** → Urea
γ-Guanidinobutyric acid	4-Aminobutanoate ← ***^Glycine amidinotransferase, mitochondrial (GATM)^*** → γ-Guanidinobutyric acid
Indole-3-acetic acid	Indole-3-acetaldehyde ← ***^Aldehyde dehydrogenase (ALDH3A2)^*** → Indole-3-acetic acid
Inosine	Adenosine ← ***^Adenosine deaminase (ADA)^*** → Inosine
Hypoxanthine	Inosine ← ***^Purine-nucleoside phosphorylase (PNP)^*** → Hypoxanthine
Xanthine	Hypoxanthine ← ***^Xanthine oxidoreductase (XOR)^*** → Xanthine
Urea	Xanthine ← ***^Xanthine oxidoreductase (XOR)^*** → Urea
Xanthosine	Xanthine ← ***^Purine-nucleoside phosphorylase (PNP)^*** → Xanthosine
1-Methylinosine	1-Methyladenosine ← ***^Adenosine deaminase (ADA)^*** → 1-Methylinosine
Cytidine	Cytidine-5′-monophosphate ← ***^5′,3′-Nucleotidase (NT5M)^*** → Cytidine
Uridine	Uridine-5′-monophosphate ← ***^5′,3′-Nucleotidase (NT5M)^*** → Uridine
Putrescine	Arginine ← ***^Arginase 1 (ARG1)^*** → Ornithine← *^Ornithine decarboxylase (ODC1)^*→ Putrescine
Phenylacetic acid	Phenylacetaldehyde ← ***^Aldehyde Dehydrogenase (ALDH3A2)^*** → Phenylacetic acid
Melatonin	Serotonin ← ***^Arylalkylamine N-acetyltransferase (AANAT)^***→ N-Acetylserotonin ← *^Acetylserotonin O-methyltransferase(ASMT)^* → Melatonin
Phenylacetylglutamine	L-glutamine ← ***^Glutamine N-phenylacetyltransferase^*** → Phenylacetylglutamine

## Abbreviations

CKD	Chronic kidney disease
AKI	Acute kidney injury
ROS	Reactive oxygen species
EUTox	European Uremic Toxins workgroup
